# The Hospital Movement in the United States

**Published:** 1906-05-19

**Authors:** 


### HOSPITAL ADMINISTRATION.

### CONSTRUCTION AND ECONOMICS.

### THE HOSPITAL MOVEMENT IN THE UNITED STATES.

### THE McLEAN HOSPITAL FOR THE INSANE AT WAVERLEY.

#### Site and Buildings.

The McLean Asylum at Waverley fulfils a very useful purpose by the admission of private patients suffering from mental diseases and subjecting them to active treatment under the most favourable conditions. The general scheme of the institution is to place the patients in relatively small households, the largest house containing not more than thirty patients at the outside. The buildings are situated on the side of a hill in the midst of well-wooded grounds, made beautiful by the wise selection and distribution of a great variety of flowering trees and shrubs of all descriptions and having woods at the back of the site. The administration block is situated in the centre of the group of buildings, and behind it are the research, clinical, and pathological departments, with the kitchen and laundry, workshops, power-house and other working departments. The houses in which the women patients are placed are situated to the right of the administration building; those for male patients being on the left. These various houses contain a few general wards, though in reality each patient has a separate bedroom; then a house for a special class of cases; then a very attractive residence for sick ladies whose means warrant their having a set of apartments to themselves; and then farther back still in the woods is the house for the reception of the more acute cases. The same general arrangement exists so far as the distribution of the male patients and their provision is concerned. There is a club, called the gymnasium, for the use of the women, and a separate club or gymnasium built for the use of the men. Each gymnasium contains a library, recreation room, billiard room, and other accommodation of an attractive kind for the use of the patients, in addition to a large and well-equipped gymnasium where all kinds of exercises are gone through under the direction of a trained instructor. Every nurse in this hospital has the advantage of a special training in the use of gymnastic apparatus of every kind, so that she may be able to teach its use and to apply it to her patients whenever occasion may arise. The courses in massage, medical gymnastics and hydrotherapy given to both male and female nurses are found to be exceedingly useful in their private work, and have increased the value of their services. Indeed, at the present day, when hydrotherapy is so much more employed than formerly, it is essential for a nurse to be able to use skilfully the procedures which are adapted to private houses, and from which exceedingly good effects can be obtained without apparatus. The demonstrations in hydrotherapy which have been given for some years at this hospital to graduates of other schools is widely appreciated. These gymnasia are the centres of many activities for treatment, for diversion, and for occupation.

#### Art Rooms and Exhibitions.

Each has an art room, large, well lighted and designed, which it is claimed diverts, educates, and refines. It is interesting to note that these art rooms which were commenced in 1885 have grown to considerable proportions mainly owing to the adoption of a suggestion made by a young lady, once a patient, while she was a resident in the hospital, to gather a loan collection for the walls of the studio and other rooms of each gymnasium building. She further suggested that certain pictures might be loaned to individual patients for the decoration of their rooms, especially when confined to their beds. As a result, the collection of pictures, comprising oils, water-colours, and photographs, has now taken a permanent place in each of the art rooms, the collections being rearranged and distributed in each April and October. Directly the hospital has benefited by the loan collections, because an increasing number of pictures is being presented to the hospital each year. The practice of loaning framed pictures to the patients, as books are loaned from the library, has been recently carried out. There are now more than fifty such pictures in circulation, and arrangements have been made for others to be made available for the same purpose. It is interesting that the young lady patient, to whose inspiration the whole of this new departure is due, on her recovery practically took charge of this branch, and has aided and sustained the movement ever since. In consequence of her efforts the collection of pictures at the McLean Hospital in the summer of 1905 numbered no less than two hundred and sixty-three pictures. The art room in the first instance was confined to the women's gymnasium, but now that the pictures are plentiful the benefits of the art gallery are shared with the men, and an art room has recently been added to the men's gymnasium. The art rooms now have a definite income, represented by the interest on a subscription fund which is steadily growing.

#### Libraries and Records.

The libraries of this hospital are a notable feature, because they contain the current literature on the subject. The records of the patients and all the clinical material and reports on treatment, with the patients' histories, are better kept and recorded than any we have heretofore seen.

#### Baths and Swedish Appliances.

There is also a bath-house used by the women in the morning and by the men in the afternoon, where various kinds of baths are available for the use of the patients under the supervision and control of skilled bath men and women who are always in attendance. In connection with the baths is a separate, well-lighted, and attractive apartment, in which are placed a number of Swedish appliances, where a skilled supervisor, acting under the direction of the medical staff, brings to bear a variety of treatment. The results are reported to be encouraging.

### A Real Hospital for Mental Cases.

It will be gathered from the foregoing description that everything about the McLean Hospital has been organised with the object of making the patients feel that they are undergoing treatment, and in consequence they should do everything in their power to co-operate with the medical staff in effecting a cure. This hospital atmosphere, as applied to every case in the McLean Hospital, has been proved by experience to exercise a most beneficial influence upon most of the inmates, and it represents a healthy development in the methods which are being brought to bear at the present time in the treatment of the mentally afflicted. The research laboratories, the chemical department, the constant taking of cases, the examination of patients, and the active clinical work which is carried on as a regular matter of routine throughout the whole of the McLean Hospital makes materially for the highest efficiency in the treatment of the insane. It secures the interest and co-operation of the patients; it keeps the medical staff alert, active, and interested in every case; and it renders a residence at the McLean Hospital, from a patient's and friend's point of view, a thing to be eminently desired. It is not surprising, therefore, that the popularity of the institution is great, and that the number of applications for admission is considerably more than can be accommodated each year. This is especially true of the women's side, fifty-four suitable cases having been declined for want of room during the year, a fact which has led the trustees to put in hand a new house so as to extend the accommodation for this class. There were 158 admissions in the year, of whom 61 were voluntary patients. The average number of patients during 1904 was 178, of which 79 were males and 99 females. The recoveries were 26.6 per cent. of the admissions and 27.6 per cent. of the dismissals. We congratulate the trustees upon the evidence we met with everywhere throughout this hospital of the wise liberality they invariably display in the provision of apparatus, in the encouragement of research, and in the spread of knowledge in relation to all matters affecting the cure and treatment of the insane.

### The Laboratory: Its Cost and Work.

The continuance of the work of the laboratory is of the first importance, but it has proved a serious tax on the resources of the hospital in the past. The staff is a large and able one, consisting, in addition to the medical superintendent, Dr. George T. Tuttle, of two assistant physicians, of a pathologist, Dr. August Hoch, with two assistants, one in pathological chemistry, Dr. Otto Folin, the other in pathological physiology, Dr. Shepard I. Franz. There are also three junior assistant physicians. A great deal of scientific work has been done at the McLean Hospital in recent years under Dr. Coles, and it is satisfactory to know that it is being extended and continued with increasing vigour by his successor, Dr. Tuttle. The purpose has been to study a great many cases together, and to collect the facts of the individual cases with each other, comparing them with the facts laid down in literature. The work, so far as it has advanced, will furnish a sound basis for further work, besides yielding practical results in developing more accurate methods in diagnosis and prognosis. The outcome of these studies under the direction of Dr. Hoch will in part be printed in an extensive publication now passing through the press. Dr. Folin's work in the chemical department laboratory is widely known and greatly appreciated. His studies in metabolism in mental diseases have led to results full of promise, and have made Dr. Folin's work widely known and appreciated by all who are interested in the subject. Such researches have only been made possible by the introduction of new analytical technique in the chemical laboratory which were developed by Dr. Folin in recent years.

The treatment includes full feeding, hydrotherapeutics, and other means for promoting sleep and improving the bodily health of those who come to the hospital in various stages of exhaustion. The intention is to provide as complete a system of physical exercise as can be devised, with a range from massage, passive and resistive movements, through medical gymnastics, Swedish movements, and use of the apparatus in the gymnasium, up to the more vigorous exercise of the various out-of-door games, which are adapted to all degrees of bodily strength, as can be done with hydro-therapeutics.

Taken as a whole, the McLean Hospital offers many points of interest to the profession and all concerned with the treatment of the insane. Dr. Tuttle has the interests of everybody at heart, and there is evidence to show that he is likely to make many material improvements of a practical character in the course of the next two years. Two great needs have been felt at this hospital. One was the need of a church, which is being met by the erection of the Samuel Eliot Memorial Chapel, which is nearly completed, and the consecration of which will render it possible to bring the amusement hall up to date and to perfect its arrangements. The need of a nurses' home, with accommodation for 90 persons, is a very pressing necessity at this hospital. At the present time the staff are scattered throughout the institution, and there is a marked want of adequate accommodation for ward-maids and members of the general staff. It is rather remarkable that the McLean Hospital, which was one of the first to train mental nurses, and which has upon its roll of graduates 509 names, should still be without a nurses' home. We beg, therefore, to draw the trustees' attention to this urgent need, and hope that the necessary sum may be voted to enable the work to be put in hand at once.

